# Secondary Endpoint Utilization and Publication Rate among Phase III Oncology Trials

**DOI:** 10.1158/2767-9764.CRC-24-0265

**Published:** 2024-08-20

**Authors:** Esther J. Beck, Alexander D. Sherry, Marcus A. Florez, Ramez Kouzy, Joseph Abi Jaoude, Timothy A. Lin, Avital M. Miller, Adina H. Passy, Gabrielle S. Kupferman, Roshal R. Patel, Fumiko Chino, Victoria Serpas Higbie, Christine M. Parseghian, Michael J. Overman, Bruce D. Minsky, Charles R. Thomas, Chad Tang, Pavlos Msaouel, Ethan B. Ludmir

**Affiliations:** 1 Division of Radiation Oncology, Department of Radiation Oncology, The University of Texas MD Anderson Cancer Center, Houston, Texas.; 2 Baylor College of Medicine, Houston, Texas.; 3 Department of Radiation Oncology, Stanford University, Stanford, California.; 4 Department of Radiation Oncology and Molecular Radiation Sciences, Johns Hopkins University School of Medicine, Baltimore, Maryland.; 5 Department of Radiation Oncology, Memorial Sloan Kettering Cancer Center, New York, New York.; 6 Division of Cancer Medicine, Department of Gastrointestinal Medical Oncology, The University of Texas MD Anderson Cancer Center, Houston, Texas.; 7 Division of Radiation Oncology, Department of Gastrointestinal Radiation Oncology, The University of Texas MD Anderson Cancer Center, Houston, Texas.; 8 Department of Radiation Oncology and Applied Sciences, Dartmouth Cancer Center, Geisel School of Medicine, Lebanon, New Hampshire.; 9 Division of Radiation Oncology, Department of Genitourinary Radiation Oncology, The University of Texas MD Anderson Cancer Center, Houston, Texas.; 10 Department of Translational Molecular Pathology, The University of Texas MD Anderson Cancer Center, Houston, Texas.; 11 Department of Investigational Cancer Therapeutics, The University of Texas MD Anderson Cancer Center, Houston, Texas.; 12 Division of Cancer Medicine, Department of Genitourinary Medical Oncology, The University of Texas MD Anderson Cancer Center, Houston, Texas.; 13 Department of Biostatistics, The University of Texas MD Anderson Cancer Center, Houston, Texas.

## Abstract

Secondary endpoints (SEP) provide crucial information in the interpretation of clinical trials, but their features are not yet well understood. Thus, we sought to empirically characterize the scope and publication rate of SEPs among late-phase oncology trials. We assessed SEPs for each randomized, published phase III oncology trial across all publications and ClinicalTrials.gov, performing logistic regressions to evaluate associations between trial characteristics and SEP publication rates. After screening, a total of 280 trials enrolling 244,576 patients and containing 2,562 SEPs met the inclusion criteria. Only 22% of trials (62/280) listed all SEPs consistently between ClinicalTrials.gov and the trial protocol. The absolute number of SEPs per trial increased over time, and trials sponsored by industry had a greater number of SEPs (median 9 vs. 5 SEPs per trial; *P* < 0.0001). In total, 69% of SEPs (1,770/2,562) were published. The publication rate significantly varied by SEP category [*X*^2^ (5, *N* = 2,562) = 245.86; *P* < 0.001]. SEPs that place the most burden on patients, such as patient-reported outcomes and translational correlatives, were published at 63% (246/393) and 44% (39/88), respectively. Trials with more SEPs were associated with lower overall SEP publication rates. Overall, our findings are that SEP publication rates in late-phase oncology trials are highly variable based on the type of SEP. To avoid undue burden on patients and promote transparency of findings, trialists should weigh the biological and clinical relevance of each SEP together with its feasibility at the time of trial design.

**Significance:**

In this investigation, we characterized the utilization and publication rates of SEPs among late-phase oncology trials. Our results draw attention to the proliferation of SEPs in recent years. Although overall publication rates were high, underpublication was detected among endpoints that may increase patient burden (such as translational correlatives and patient-reported outcomes).

## Introduction

Secondary endpoints (SEP) are trial outcome measures that address important complementary questions to the primary endpoint (PEP); these SEPs may be used to assess treatment efficacy, patient symptoms, correlative translational analyses, and more (1). In oncology trials, SEPs—particularly translational correlatives—often provide rich, valuable information critical to the interpretation of the trial and the PEP, and may lead to the development of new trials and research directions (2).

Whereas there has been much focus on the selection, validity, and transparency of PEPs in oncology trials, relatively less attention has been given to SEPs (3–6). The nature and number of SEPs have an impact on the research burden placed on clinical research infrastructure and especially on patients, who are often asked to donate their time and specimens to advance medical knowledge. Despite the direct impact of SEPs on patients and the overall trial interpretation, the scope and reporting of SEPs across oncology are poorly understood. Selective nonreporting and underpublication of PEPs have been shown to be particularly problematic in oncology trials (7–12). Previous studies have shown high variability in thoroughness and compliance with mandatory reporting requirements through trial registries (13, 14). Transparency in reporting of endpoints is further complicated by the fact that study protocols and their amendments are often unpublished, inaccessible, incomplete, or redacted (15–17). Thus, we sought to investigate trends in the frequency, characteristics, and reporting of SEPs in late-phase oncology trials.

## Materials and Methods

**Figure 1:**
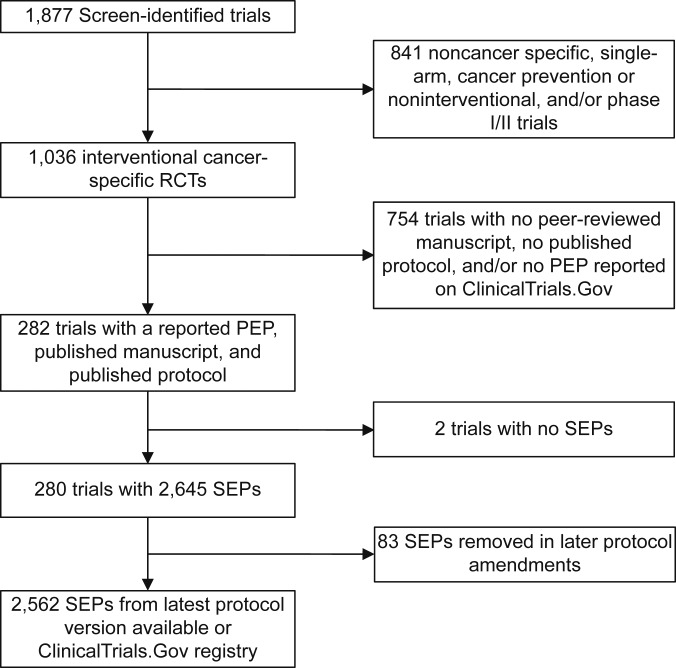
Flow diagram of clinical trial screening and SEP inclusion criteria. Cancer-specific, phase III randomized clinical trials (RCTs) were found using ClinicalTrials.Gov in February 2020. SEPs were found using ClinicalTrials.Gov, the protocol, and all associated publications.

We screened ClinicalTrials.gov from inception through February 2020 for phase III cancer-specific interventional randomized controlled trials, as previously described (17). Trials were included if the study (i) had published an article with its PEP results through 2020, (ii) had an available protocol, and (iii) contained at least one SEP (Fig. 1). We found published articles via both ClinicalTrials.gov and PubMed searches using National Clinical Trial numbers and, if necessary, key words related to the study. Institutional review board approval was waived because of the public availability of data. This study complied with STROBE guidelines (18).

For each included trial, we manually collected SEPs from ClinicalTrials.gov, all available protocol versions, and published articles. The availability and completeness of protocols were also manually validated. SEPs were defined narrowly and only used if labeled specifically as SEPs, outcome measures, or variables, depending on the trial’s preferred language. By contrast, secondary objectives and tertiary or exploratory endpoints were not independently considered SEPs. Moreover, SEPs that were removed in later protocol amendments were not included for the purposes of this study.

We reviewed all available published articles to track data for each SEP, with trial publications queried between June and October 2023. We only recorded a SEP as published once it had reached maturity. If a SEP was discussed but no data were listed or available, it was not considered as having been published. We also considered data reported under the “Results” section for a given trial on ClinicalTrials.gov. SEPs were classified into categories. Disease-related outcomes (DRO) encompassed all tumor- and survival-related outcomes. Patient-reported outcomes (PRO) were derived from patients’ answers to questionnaires that typically assessed aspects of their quality of life. Toxicity endpoints covered provider-evaluated adverse events. Translational correlatives included all biomarker, imaging, and biological sample analyses. Pharmacokinetic endpoints evaluated drug metabolism and kinetics. Economic endpoints measured medical resource usage and financial toxicities.

We defined SEPs as having been published when their data were found in a peer-reviewed manuscript, inclusive of data published in supplementary materials with or without interpretation. SEPs with data that were not published, but uploaded in full on ClinicalTrials.gov were defined as reported but not published. To account for variability in publication rate for SEPs collected from different sources, we ran a sensitivity analysis restricting the evaluated SEPs to only those that were (i) listed on both ClinicalTrials.gov and the latest version of the protocol and (ii) from trials with multiple protocol versions. These SEPs had the highest fidelity and were the most consistently acknowledged endpoints associated with each trial.

Continuous variables were summarized by median and IQR and categorical variables by frequency. Mann–Whitney *U*-tests were used to detect differences in the numbers of SEPs by trial sponsorship; if trials were sponsored by both industry and cooperative groups, they were grouped in both categories. Trial-level characteristics and the rate of SEP publication were first evaluated using ordinary-least squares regression. Subsequently, the SEP publication rate for each trial was dichotomized into optimal publication rate (>75%) and suboptimal publication rate (≤75%), which represented 49% and 51% of trials in the dataset, respectively. We then employed binary logistic regression to explore associations and calculate ORs. To account for the potential influence of confounding variables, we then adjusted these associations using multivariable binary logistic regression. Confounding variables were identified by mapping causal relationships on a directed acyclic graph using DAGitty (Supplementary Fig. S1; ref. 19). All tests were two-sided, confidence intervals (CI) were reported at 95%, and *α* was set *a priori* at 0.05. Statistical analyses were performed using SPSS v24 (IBM) and SAS v9.4. Plots were created using Prism v10 (GraphPad).

### Data availability

Research data are stored in an institutional repository and will be shared upon reasonable request to the corresponding author.

## Results

A total of 280 trials enrolling 244,576 patients with publication dates ranging from 2010 to 2023 met the inclusion criteria for this study (Fig. 1). Whereas all included trials had an available trial protocol, 55% of studies (153/280) provided more than one protocol or a summary of amendments (Table 1). There was a median follow-up time of 8 years per trial after the primary publication to the end of data capture (IQR: 6–10 years).

**Table 1:** Characteristics of selected phase III randomized controlled trials in oncology

Trial characteristic	Frequency, *N* (%)
Industry sponsorship	214 (76%)
Cooperative group sponsorship	96 (34%)
Disease site	
Breast	49 (18%)
Gastrointestinal	27 (10%)
Genitourinary	39 (14%)
Head and neck	11 (4%)
Hematologic	55 (20%)
Thoracic	39 (14%)
Other	60 (21%)
Treatment modality^a^	
Systemic therapy	240 (86%)
Radiotherapy	14 (5%)
Surgery	2 (1%)
Supportive care	24 (9%)
Disease setting	
Upfront	161 (58%)
Relapsed/refractory	119 (42%)
FDA approval	115 (41%)
PEP met	171 (61%)
Multiple protocols available^b^	153 (55%)

a Treatment modality was decided by the primary intervention for each trial, whether systemic (including chemotherapies, immunotherapies, and other systemic agents), surgical, radiotherapies, or supportive care trials (aimed at alleviating the toxic effects of disease or treatment).

b Of the 280 trials, 153 had more than one protocol version available with unredacted sections in regard to SEPs or provided a summary of amendments.

**Figure 2:**
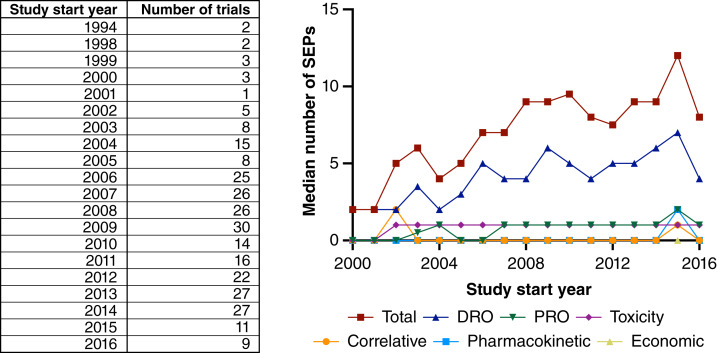
Trends in the number of SEPs and SEP categories over time. The median overall number of SEPs is represented for each year, as well as the median numbers for each SEP category. For ease of visualization, the seven trials before 2000 were not included in the figure.

Across the 280 trials examined, there were a total of 2,562 SEPs. A median of eight SEPs was found per trial (IQR: 5–12). Notably, seven trials had 25 or more SEPs, with the highest number observed being 48 SEPs in a single trial. Most of the SEPs (66%; 1,700/2,562) were documented in both ClinicalTrials.gov and the respective trial protocol. The remaining SEPs were recorded exclusively in one of three places: only on ClinicalTrials.gov, only in the protocol, or only in a publication, as detailed in Supplementary Table S1. Only 22% of trials (62/280) listed all their SEPs consistently across both ClinicalTrials.gov and the last available version of the protocol. The absolute number of SEPs per trial increased over time (*β* = 0.36; *P* < 0.0001; Fig. 2). The number of SEPs was associated with trial sponsorship, with an increased median number of SEPs per trial for industry-sponsored studies versus nonindustry-sponsored studies (median 9 vs. 5 SEPs per study; *P* < 0.0001).

**Figure 3:**
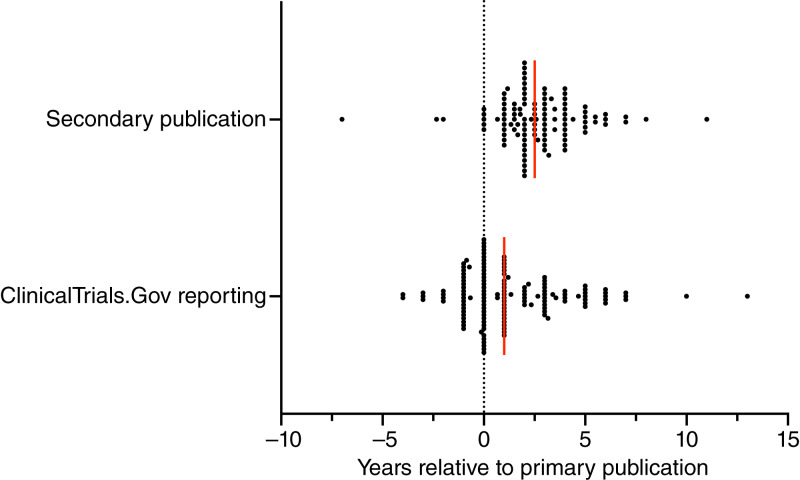
Time course of secondary publication and ClinicalTrials.Gov reporting relative to primary publication. Red lines represent the median years to event, and dots represent individual trials. Dotted line represents the year the primary publication was released. SEPs were reported on ClinicalTrials.Gov a median of 1 year after primary publication (IQR: 0–3 years); secondary publications were released a median of 2.5 years after primary publication (IQR: 1.5–4 years).

Overall, 69% of SEPs (1,770/2,562) were ever published. Half of the SEPs (50%, 1,268/2,562) were published in the main text of the primary article. The remaining published SEPs were distributed among the supplement of the primary article (7%, 183/2,562), the main text of a secondary publication (12%, 300/2,562), and the supplement of a secondary publication (1%, 19/2,562; Table 2). Secondary articles with SEP results were published a median of 2.5 years after the primary publication (IQR: 1.5–4; Fig. 3). Half of all trials (144/280) published more than 75% of their SEPs. The publication rate significantly varied by SEP category [*X*^2^ (5, *N* = 2,562) = 245.86; *P* < 0.001]. DROs and toxicity endpoints were published at the highest rates of 75% (1,137/1,514) and 78% (309/396), respectively, whereas pharmacokinetics and economic measures were the lowest at 24% (37/155) and 13% (2/16; Table 2), respectively. Sixty-three percent of all PROs were published; of the 169 trials with at least one PRO endpoint, 52% (88/169) published all their PROs, and 28% (48/169) published none of them (Supplementary Table S2). Translational correlatives were 44% (39/88) published overall, with 36% (15/42) of those based on blood testing published, compared with 52% (16/31) of those requiring tissue samples or bone marrow aspirations (Supplementary Table S3).

**Table 2:** Comparison of publication rates by SEP category

SEP	*N* ^a^	Published, *N* (%)	Primary article, *N* (%)^b^	Supplement of primary article, *N* (%)^b^	Secondary article, *N* (%)^c^	Supplement of secondary article, *N* (%)^c^
Total	2,562	1,770 (69%)	1,268 (50%)	183 (7%)	300 (12%)	19 (1%)
Category^d^						
DRO	1,514	1,137 (75%)	885 (59%)	102 (7%)	149 (10%)	1 (0.1%)
PRO	393	246 (63%)	95 (24%)	53 (14%)	80 (20%)	18 (5%)
Toxicity	396	309 (78%)	261 (65%)	21 (5%)	27 (7%)	0 (0%)
Correlatives	88	39 (44%)	13 (15%)	3 (3%)	23 (26%)	0 (0%)
Pharmacokinetics	155	37 (24%)	13 (8%)	4 (3%)	20 (13%)	0 (0%)
Economic	16	2 (13%)	1 (6%)	0 (0%)	1 (6%)	0 (0%)

a *N* is representative of all endpoints included in this trial, not just those published and therefore represented in this table.

b The primary article was the publication containing the final results of the PEP analysis. All endpoints that had data inside the body, figures, or tables of the article were considered to be in the text. Any SEPs with data located within supplementary figures or tables were considered to be in the supplement.

c The secondary article was any article containing results beyond the PEP analysis, whether it was published before or after the primary article. All endpoints that had data inside the body, figures, or tables of the article were considered to be found in the text. Any SEPs with data located within supplementary figures or tables were considered to be in the supplement.

d SEPs were stratified by category to examine the differences in publication between different types of SEPs.

Trials with greater numbers of SEPs were more likely to underpublish their SEPs, defined by a publication rate of 75% or less (OR 1.15; 95% CI, 1.09–1.22; *P* < 0.0001). This association persisted after adjustment for confounders (adjusted OR 1.16; 95% CI, 1.09–1.22; *P* < 0.0001; Supplementary Table S4A). Publication also seemed to be related to DRO SEPs; trials with a greater percentage of DRO endpoints were less likely to underpublish, even after adjustment for number of SEPs per trial (adjusted OR 0.30; 95% CI, 0.11–0.85; *P* = 0.02; Supplementary Table S4B). Other trial-level factors did not seem to be strongly associated with underpublication (Supplementary Table S5A–S5H). Lastly, disease setting (upfront vs. relapsed/refractory; OR 0.90; 95% CI, 0.56–1.45; *P* = 0.7) and primary publication year (OR 0.97; 95% CI, 0.88–1.07; *P* = 0.5) were also not associated with the SEP publication rate.

Owing to the heterogeneity in SEPs listed between the registry and the protocol, the analysis was repeated looking at the highest fidelity SEPs: only those that were (i) listed on both ClinicalTrials.gov and the protocol and (ii) from trials with multiple protocol versions. These 1,068 SEPs were the most consistently acknowledged in association with their trials, even after protocol amendments. In this sensitivity analysis, 74% of SEPs (794/1,068) were published, and 59% of trials (60/147) published greater than 75% of their SEPs (Supplementary Table S6). The number of SEPs remained associated with underpublication (adjusted OR 1.27; 95% CI, 1.14–1.41; *P* < 0.0001; Supplementary Table S7).

Whereas 31% (792/2,562) of total SEPs were not published, 19% of SEPs (491/2,562) were unpublished but had data reported on ClinicalTrials.gov (Table 3). SEP data were reported on ClinicalTrials.gov a median of 1 year after the primary publication (IQR: 0–3 years) and a median of 1.5 years before secondary publications containing SEP results (Fig. 3). For 8% of SEPs (203/2,562), result data were never reported on ClinicalTrials.gov or published, and no justification was provided as to their unavailability (Table 3). Of all economic and translational correlative SEPs, 69% (11/16) and 26% (23/88), respectively, were missing, never having been published or reported.

**Table 3:** ClinicalTrials.Gov reporting and missing data among unpublished endpoints

SEPs	*N* ^a^	ClinicalTrials.Gov reported, *N* (%)^b^	Excused, *N* (%)^c^	Missing, *N* (%)^d^
Total	2,562	491 (19%)	98 (4%)	203 (8%)
SEPs by detection method^e^				
ClinicalTrials.Gov and protocol	1,700	294 (17%)	86 (5%)	61 (4%)
ClinicalTrials.Gov only	474	191 (40%)	10 (2%)	25 (5%)
Protocol only	325	5 (2%)	2 (1%)	107 (33%)
Publication only	63	1 (2%)	0 (0%)	10 (16%)
SEPs by category^f^				
DRO	1,514	205 (14%)	81 (5%)	91 (6%)
PRO	393	104 (27%)	9 (2%)	34 (9%)
Toxicity	396	68 (17%)	2 (1%)	17 (4%)
Correlatives	88	22 (25%)	4 (5%)	23 (26%)
Pharmacokinetics	155	89 (57%)	2 (1%)	27 (17%)
Economic	16	3 (19%)	0 (0%)	11 (69%)

a *N* is representative of all endpoints included in this trial, not just those unpublished and therefore represented in this table.

b Endpoints that were not published but had their complete associated data uploaded onto the ClinicalTrials.Gov registry were considered reported.

c Endpoints were considered excused if they were not published or reported, but reasoning for the data’s unavailability was provided on ClinicalTrials.Gov or an associated publication.

d Endpoints that were not published, reported on ClinicalTrials.Gov, or excused, representing SEPs originally associated within a trial but with data that ultimately were never made available. Forty-eight of these endpoints were acknowledged by the authors in a publication but contained no justification as to why the data were not yet available.

e SEPs were stratified using the detection method used to originally locate them.

f SEPs were stratified by category to examine the differences in publication, reporting, and missing data between different types of SEPs.

## Discussion

In this large-scale analysis of SEPs among phase III oncology clinical trials, the number of SEPs was shown to have considerably increased over time, and the majority of SEPs were shown to be published. However, SEP underpublication is particularly prominent among PROs and translational endpoints. SEP underpublication may present ethical challenges considering patient burden associated with obtaining biospecimens for correlative analyses, as well as the time commitment required for SEP compliance (i.e., PROs; refs. 20, 21). The number of SEPs seems related to underpublication, suggesting that the increasing numbers of SEPs per trial are prohibitive for reliable publication reporting. To appropriately respect the burden placed on patients, as well as limit multiplicity concerns, trialists should thoughtfully weigh the feasibility and practicality of SEPs in conjunction with clinical relevance toward key research questions.

Although other studies have focused on more limited sets of endpoints, to the best of our knowledge, this is the first and only comprehensive analysis of all SEPs across a large cohort of phase III oncology trials. Defining SEPs for each trial was challenging, as our thorough manual review found that SEPs were inconsistently recorded across available protocols and the ClinicalTrials.gov registry, in line with previous analyses (22, 23). Thus, the manually validated diversity of sources used both to initially extract SEPs and track publication data contributed to a more in-depth understanding of the trial landscape, detecting inconsistencies in the handling of SEP data that would not have been possible had only one source been used. Notably, although many unpublished SEPs did ultimately have data reported on ClinicalTrials.gov, ClinicalTrials.gov results were presented without explanation or analysis—and at times, without inferential statistical testing. Therefore, it presents difficulties in interpretation for patients and physicians who are not content matter experts (21).

Our analysis also raised questions about the underpublication of particular data types, especially PROs and correlatives. PROs are crucial to providing the patient’s perspective on tolerability and toxicity and add valuable information beyond physician assessment of adverse events; however, the completion of lengthy questionnaires can be time-consuming and distressing to patients (24, 25). Survey fatigue from lengthy questionnaires has also been shown to increase respondent attrition rates and compromise response quality (26, 27). Translational correlatives often require the collection of biological specimens from patients and may be associated with painful and invasive procedures obtained outside the context of routine clinical care. Given the burden such SEPs may place on patients, trials should particularly endeavor to publish these data in a timely manner to aid in the interpretation of the PEP and other SEPs (20, 21).

There are several key limitations to this study. To capture the full range of each trial’s SEPs, we examined only trials with published online protocols, but low protocol availability rates among oncology trials limited our overall sample size (15). Incomplete protocols and lack of multiple protocol versions may also limit the transparency of the final confirmed SEPs per trial, despite our comprehensive examination of publicly available data across the trial protocols, publications, and ClinicalTrials.gov. To account for the standard study procedure of editing SEPs after initial trial design, we chose not to examine SEPs that were removed in later protocol amendments. However, these may have already been evaluated on patients, thus contributing further to the effect size of underpublication. Additionally, data that were published through nonpeer-reviewed mechanisms such as lay press or company websites were not examined under the scope of our study, although such data would potentially be available to patients. Further follow-up time could lead to higher rates of SEP publication as data matures and secondary articles are released, although a minimum of 8 years after the study start year was provided for each trial.

In summary, this comprehensive examination of the oncology clinical trial landscape highlights the imperative of SEP publication and transparency across all endpoint types. At the time of trial design, SEPs should be thoughtfully selected to those that are biologically plausible and supported by other clinical evidence or rationales, while being conscientious of the burden on patients. To truly promote transparency surrounding these endpoints, trials should endeavor to publish complete protocols and amendments, ideally in the form of first and last or summary of changes. Finally, all prespecified endpoints should be published on a reasonable timeline; when that is not possible, the rationale for nonreporting should be provided.

## Supplementary Material

Supplemental Figure S1 Structural casual model of the relationship between the number of SEPs, confounding variables, and the percent of SEPs published. Orange represents the exposure of interest (number of SEPs), yellow represents the outcome of interest (percent of SEPs published), and the red arrow indicates the causal path. Green circles indicate confounders, blue circles indicate non-confounding ancestors of the exposure and outcome. Black arrows represent biasing pathway.

Supplemental Table S1 Comparison of publication rates by SEP detection method.

Supplemental Table S2 Distribution of the percentage of PROs published per trial.

Supplemental Table S3 Distribution of the types of correlatives and their respective publication rates.

Supplemental Table S4 Full multivariable model evaluating the association between significant trial-level factors and the percentage of SEPs published.

Supplemental Table S5 Full multivariable model evaluating the association between nonsignificant trial-level factors and the percentage of SEPs published.

Supplemental Table S6 Comparison of publication and reporting between the overall dataset and the sensitivity analysis restricting SEPs to only those from both the protocol and ClinicalTrials.Gov, from trials with multiple protocols available.

Supplemental Table S7 Full multivariable model evaluating the association between the number of SEPs and the percentage of SEPs published among sensitivity analysis SEPs from both the protocol and ClinicalTrials.Gov, from trials with multiple protocols available.
