# Supplementary material for: Secondary Endpoint Utilization and Publication Rate among Phase III Oncology Trials
Source: Cancer Res Commun. 2024 Aug 20;4(8):2183–8. doi: 10.1158/2767-9764.CRC-24-0265 (PMC11333994; doi:10.1158/2767-9764.CRC-24-0265)
Supplement: Supplemental Figure S1 — Structural casual model of the relationship between the number of SEPs, confounding variables, and the percent of SEPs published. Orange represents the exposure of interest (number of SEPs), yellow represents the outcome of interest (percent of SEPs published), and the red arrow indicates the causal path. Green circles indicate confounders, blue circles indicate non-confounding ancestors of the exposure and outcome. Black arrows represent biasing pathway. [file crc-24-0265_supplemental_figure_s1_supps1.docx]

**Supplementary Figure S1.** Structural casual model of the relationship between the number of SEPs, confounding variables, and the percent of SEPs published. Orange represents the exposure of interest (number of SEPs), yellow represents the outcome of interest (percent of SEPs published), and the red arrow indicates the causal path. Green circles indicate confounders, blue circles indicate non-confounding ancestors of the exposure and outcome. Black arrows represent biasing pathway.

Abbreviations: SEP, Secondary Endpoint; DRO, Disease-Related Outcome.
