# Supplementary material for: Secondary Endpoint Utilization and Publication Rate among Phase III Oncology Trials
Source: Cancer Res Commun. 2024 Aug 20;4(8):2183–8. doi: 10.1158/2767-9764.CRC-24-0265 (PMC11333994; doi:10.1158/2767-9764.CRC-24-0265)
Supplement: Supplemental Table S1 — Comparison of publication rates by SEP detection method. [file crc-24-0265_supplemental_table_s1_supps1.docx]

**Suppemental Table S1**. Comparison of publication rates by SEP detection method.

| **Stratification^a^** | ***N*** | **Published, *N* (%)** | **Text of primary paper, *N* (%) ^b^** | **Supplement of primary paper, *N* (%) ^b^** | **Text of secondary paper, *N* (%)** ^c^ | **Supplement of secondary paper, *N* (%)** ^c^ |
| --- | --- | --- | --- | --- | --- | --- |
| Total | 2562 | 1770 (69%) | 1268 (50%) | 183 (7%) | 300 (12%) | 19 (1%) |
| ClinicalTrials.Gov and Protocol | 1700 | 1259 (74%) | 886 (52%) | 141 (8%) | 218 (13%) | 14 (1%) |
| ClinicalTrials.Gov | 474 | 248 (52%) | 184 (39%) | 20 (4%) | 40 (8%) | 4 (1%) |
| Protocol | 325 | 211 (65%) | 155 (48%) | 16 (5%) | 39 (12%) | 1 (0.3%) |
| Paper | 63 | 52 (83%) | 43 (68%) | 6 (10%) | 3 (5%) | 0 (0%) |

Abbreviations: SEP, Secondary Endpoint

^a^ Secondary endpoints were stratified by the detection method used to originally locate them.

^b^ The primary paper was the publication containing the final results of the primary endpoint analysis. All endpoints that had data inside the body, figures, or tables of the article were considered to be in the text. Any SEPs with data located within supplementary figures or tables were considered to be in the supplement.

^c^ The secondary paper was any paper containing results beyond the primary endpoint analysis, whether it was published before or after the primary paper. All endpoints that had data inside the body, figures, or tables of the article were considered to be found in the text. Any SEPs with data located within supplementary figures or tables were considered to be in the supplement.
