# Supplementary material for: Secondary Endpoint Utilization and Publication Rate among Phase III Oncology Trials
Source: Cancer Res Commun. 2024 Aug 20;4(8):2183–8. doi: 10.1158/2767-9764.CRC-24-0265 (PMC11333994; doi:10.1158/2767-9764.CRC-24-0265)
Supplement: Supplemental Table S2 — Distribution of the percentage of PROs published per trial. [file crc-24-0265_supplemental_table_s2_supps2.docx]

**Supplemental Table S2.** Distribution of the percentage of PROs published per trial.

| **Percentage of PROs published by trial** | ***N* (%)** |
| --- | --- |
| 0% | 48 (28%) |
| 1 - 25% | 3 (2%) |
| 26 - 50% | 16 (10%) |
| 50 - 75% | 11 (7%) |
| 75 - 99% | 3 (2%) |
| 100% | 88 (52%) |

Abbreviations: PRO, Patient-Reported Outcome
