# Supplementary material for: Secondary Endpoint Utilization and Publication Rate among Phase III Oncology Trials
Source: Cancer Res Commun. 2024 Aug 20;4(8):2183–8. doi: 10.1158/2767-9764.CRC-24-0265 (PMC11333994; doi:10.1158/2767-9764.CRC-24-0265)
Supplement: Supplemental Table S3 — Distribution of the types of correlatives and their respective publication rates. [file crc-24-0265_supplemental_table_s3_supps3.docx]

**Supplemental Table S3.** Distribution of the types of correlatives and their respective publication rates.

| **Correlative Type** | ***N*** | **Published, *N* (%)** |
| --- | --- | --- |
| Blood | 42 | 15 (36%) |
| Tissue | 30 | 16 (53%) |
| Multiple sources ^a^ | 9 | 6 (67%) |
| Not Specified ^b^ | 4 | 1 (25%) |
| Bone Marrow Aspiration | 1 | 0 (0%) |
| DNA Sample Kit | 1 | 0 (0%) |
| Scan | 1 | 1 (100%) |

^a^ Several endpoints called for the use of correlative data from multiple sources. These endpoints were separated to prevent overlap among the independent correlative types.

^b^ Several trials did not explicitly state the method of collection of their samples for correlative endpoints in the available protocol versions.
