# Supplementary material for: Secondary Endpoint Utilization and Publication Rate among Phase III Oncology Trials
Source: Cancer Res Commun. 2024 Aug 20;4(8):2183–8. doi: 10.1158/2767-9764.CRC-24-0265 (PMC11333994; doi:10.1158/2767-9764.CRC-24-0265)
Supplement: Supplemental Table S4 — Full multivariable model evaluating the association between significant trial-level factors and the percentage of SEPs published. [file crc-24-0265_supplemental_table_s4_supps4.docx]

**Supplemental Table S4**. Full multivariable model evaluating the association between significant trial-level factors and the percentage of SEPs published.

A) Multivariable model for the number of SEPs per trial

| **Variable** | **aOR** | **95% CI** | ***P*** |
| --- | --- | --- | --- |
| *Factor of Interest* |  |  |  |
| Number of SEPs | 1.16 | 1.09 to 1.22 | <.0001 |
| *Confounders* |  |  |  |
| Industry Sponsorship | 1.11 | 0.45 to 2.74 | 0.8 |
| Cooperative Group Sponsorship | 1.39 | 0.64 to 3.05 | 0.4 |
| Treatment Type – Systemic ^1^ | 1.54 | 0.67 to 3.54 | 0.3 |

B) Multivariable model for the percentage of DRO SEPs per trial.

| **Variable** | **aOR** | **95% CI** | ***P*** |
| --- | --- | --- | --- |
| *Factor of Interest* |  |  |  |
| Percent of DRO SEPs | 0.30 | 0.11 to 0.85 | 0.02 |
| *Confounders* |  |  |  |
| Cooperative Group Sponsorship | 1.30 | 0.60 to 2.825 | 0.5 |
| Industry Sponsorship | 1.55 | 0.64 to 3.773 | 0.3 |
| Number of SEPs | 1.14 | 1.08 to 1.206 | <.0001 |

Abbreviations: SEP, Secondary Endpoint; aOR, adjusted Odds Ratio; CI, Confidence Interval

^1^ Treatment modality was decided by the primary intervention for each trial, whether systemic (including chemotherapies, immunotherapies, and other systemic agents), surgical, radiotherapies, or supportive care trials (aimed at alleviating the toxic effects of disease or treatment). Because of the uneven distribution among trials in this dataset, treatment type was evaluated in the model as systemic vs non-systemic.
