# Supplementary material for: Secondary Endpoint Utilization and Publication Rate among Phase III Oncology Trials
Source: Cancer Res Commun. 2024 Aug 20;4(8):2183–8. doi: 10.1158/2767-9764.CRC-24-0265 (PMC11333994; doi:10.1158/2767-9764.CRC-24-0265)
Supplement: Supplemental Table S5 — Full multivariable model evaluating the association between nonsignificant trial-level factors and the percentage of SEPs published. [file crc-24-0265_supplemental_table_s5_supps5.docx]

**Supplemental Table S5**. Full multivariable model evaluating the association between nonsignificant trial-level factors and the percentage of SEPs published.

A) Multivariable model evaluating by trials that met their primary endpoint;

| **Variable** | **aOR** | **95% CI** | ***P*** |
| --- | --- | --- | --- |
| *Factor of Interest* |  |  |  |
| Primary Endpoint Met | 1.14 | 0.67 to 1.93 | 0.6 |
| *Confounders* |  |  |  |
| Number of SEPs | 1.15 | 1.09 to 1.22 | <0.0001 |
| Percent of DRO SEPs | 0.32 | 0.12 to 0.86 | 0.02 |
| Primary Publication Year ^1^ | 0.92 | 0.83 to 1.02 | 0.1 |

B) Multivariable model evaluating by trials that contained surrogate primary endpoints;

| **Variable** | **aOR** | **95% CI** | ***P*** |
| --- | --- | --- | --- |
| *Factor of Interest* |  |  |  |
| Primary Endpoint Type – Surrogate | 1.26 | 0.77 to 2.06 | 0.4 |
| *Confounders* |  |  |  |
| Cooperative Group Sponsorship | 1.12 | 0.54 to 2.34 | 0.8 |
| Industry Sponsorship | 1.46 | 0.62 to 3.44 | 0.4 |
| Treatment Type – Systemic Therapy ^2^ | 1.18 | 0.53 to 2.63 | 0.7 |

C) Multivariable model evaluating by trials that represented systemic therapies;

| **Variable** | **aOR** | **95% CI** | ***P*** |
| --- | --- | --- | --- |
| *Factor of Interest* |  |  |  |
| Treatment Type – Systemic Therapy ^2^ | 1.26 | 0.57 to 2.77 | 0.6 |
| *Confounders* |  |  |  |
| Cooperative Group Sponsorship | 1.10 | 0.53 to 2.30 | 0.8 |
| Industry Sponsorship | 1.48 | 0.63 to 3.48 | 0.4 |

D) Multivariable model evaluating by trial enrollment size;

| **Variable** | **aOR** | **95% CI** | ***P*** |
| --- | --- | --- | --- |
| *Factor of Interest* |  |  |  |
| Enrollment Size | 1.00 | 1.00 to 1.00 | 0.9 |
| *Confounders* |  |  |  |
| Industry Sponsorship | 1.60 | 0.70 to 3.63 | 0.3 |
| Cooperative Group Sponsorship | 1.09 | 0.52 to 2.28 | 0.8 |

E) Multivariable model evaluating by cooperative group sponsored trials;

| **Variable** | **aOR** | **95% CI** | ***P*** |
| --- | --- | --- | --- |
| *Factor of Interest* |  |  |  |
| Cooperative Group Sponsorship | 0.79 | 0.48 to 1.30 | 0.4 |

F) Multivariable model evaluating by industry sponsored trials;

| **Variable** | **aOR** | **95% CI** | ***P*** |
| --- | --- | --- | --- |
| *Factor of Interest* |  |  |  |
| Industry Sponsorship | 1.50 | 0.86 to 2.62 | 0.2 |

G) Multivariable model evaluating by trials that met their accrual goals;

| **Variable** | **aOR** | **95% CI** | ***P*** |
| --- | --- | --- | --- |
| *Factor of Interest* |  |  |  |
| Accrual Goals Met | 1.04 | 0.49 to 2.21 | 0.9 |
| *Confounders* |  |  |  |
| Cooperative Group Sponsorship | 1.28 | 0.58 to 2.814 | 0.5 |
| Industry Sponsorship | 1.19 | 0.49 to 2.871 | 0.7 |
| Number of SEPs | 1.16 | 1.10 to 1.227 | <0.0001 |
| Primary Publication Year ^1^ | 0.93 | 0.84 to 1.030 | 0.2 |
| Primary Endpoint Type – Surrogate | 1.19 | 0.71 to 1.997 | 0.5 |

H) Multivariable model evaluating by a trial’s primary publication year.

| **Variable** | **aOR** | **95% CI** | ***P*** |
| --- | --- | --- | --- |
| *Factor of Interest* |  |  |  |
| Primary Publication Year ^1^ | 0.83 | 1.02 to 0.11 | 0.1 |
| *Confounders* |  |  |  |
| Number of SEPs | 1.15 | 1.09 to 1.218 | <0.0001 |
| Primary Endpoint Met | 1.14 | 0.67 to 1.927 | 0.6 |
| Percent of DRO SEPs | 0.32 | 0.12 to 0.857 | 0.02 |

Abbreviations: SEP, Secondary Endpoint; DRO, Disease-Related Outcome; aOR, adjusted Odds Ratio; CI, Confidence Interval

^1^ The primary publication year was the year that the paper reporting on the results of the primary endpoint analysis was published.

^2^ Treatment modality was decided by the primary intervention for each trial, whether systemic (including chemotherapies, immunotherapies, and other systemic agents), surgical, radiotherapies, or supportive care trials (aimed at alleviating the toxic effects of disease or treatment). Because of the uneven distribution among trials in this dataset, treatment type was evaluated in the model as systemic vs non-systemic.
