# Supplementary material for: Secondary Endpoint Utilization and Publication Rate among Phase III Oncology Trials
Source: Cancer Res Commun. 2024 Aug 20;4(8):2183–8. doi: 10.1158/2767-9764.CRC-24-0265 (PMC11333994; doi:10.1158/2767-9764.CRC-24-0265)
Supplement: Supplemental Table S6 — Comparison of publication and reporting between the overall dataset and the sensitivity analysis restricting SEPs to only those from both the protocol and ClinicalTrials.Gov, from trials with multiple protocols available. [file crc-24-0265_supplemental_table_s6_supps6.docx]

**Supplemental Table S6**. Comparison of publication and reporting between the overall dataset and the sensitivity analysis restricting SEPs to only those from both the protocol and ClinicalTrials.Gov, from trials with multiple protocols available.

|  | **Overall Dataset, *N* (%)** | **Sensitivity Analysis, *N* (%)** |
| --- | --- | --- |
| *Total* *SEPs* | *2562* | *1068* |
| Published | 1770 (69%) | 794 (74%) |
| Reported on ClinicalTrials.Gov ^a^ | 491 (19%) | 187 (18%) |
| Excused ^b^ | 98 (4%) | 46 (4%) |
| Missing ^c^ | 203 (8%) | 41 (4%) |

^a^ Endpoints that were not published but had their complete associated data uploaded onto the ClinicalTrials.Gov registry were considered reported.

^b^ Endpoints were considered excused if they were not published or reported, but reasoning for the data’s unavailability was provided on ClinicalTrials.Gov or an associated publication

^c^ Missing endpoints were never published, reported, or excused, representing endpoints originally associated within a trial but without available data.
