# Supplementary material for: Secondary Endpoint Utilization and Publication Rate among Phase III Oncology Trials
Source: Cancer Res Commun. 2024 Aug 20;4(8):2183–8. doi: 10.1158/2767-9764.CRC-24-0265 (PMC11333994; doi:10.1158/2767-9764.CRC-24-0265)
Supplement: Supplemental Table S7 — Full multivariable model evaluating the association between the number of SEPs and the percentage of SEPs published among sensitivity analysis SEPs from both the protocol and ClinicalTrials.Gov, from trials with multiple protocols available. [file crc-24-0265_supplemental_table_s7_supps7.docx]

**Supplemental Table S7**. Full multivariable model evaluating the association between the number of SEPs and the percentage of SEPs published among sensitivity analysis SEPs from both the protocol and ClinicalTrials.Gov, from trials with multiple protocols available.

| **Variable** | **aOR** | **95% CI** | ***P*** |
| --- | --- | --- | --- |
| *Factor of Interest* |  |  |  |
| Number of SEPs | 1.27 | 1.14 to 1.41 | <0.0001 |
| *Confounders* |  |  |  |
| Industry Sponsorship | 0.84 | 0.21 to 3.26 | 0.8 |
| Cooperative Group Sponsorship | 1.65 | 0.53 to 5.12 | 0.4 |
| Treatment Type – Systemic ^a^ | 0.59 | 0.16 to 2.23 | 0.4 |

Abbreviations: SEP, secondary outcome measure; aOR, adjusted odds ratio; CI, confidence interval

^a^ Treatment modality was decided by the primary intervention for each trial, whether systemic (including chemotherapies, immunotherapies, and other systemic agents), surgical, radiotherapies, or supportive care trials (aimed at alleviating the toxic effects of disease or treatment). Because of the uneven distribution among trials in this dataset, treatment type was evaluated in the model as systemic vs non-systemic.
